# Near-Complete Genome Sequence of Ralstonia solanacearum T523, a Phylotype I Tomato Phytopathogen Isolated from the Philippines

**DOI:** 10.1128/MRA.01048-18

**Published:** 2018-09-27

**Authors:** Andrew D. Montecillo, Asuncion K. Raymundo, Irene A. Papa, Genevieve Mae B. Aquino, Arian J. Jacildo, Paul Stothard, Albert Remus R. Rosana

**Affiliations:** a Microbiology Division, Institute of Biological Sciences, University of the Philippines Los Baños, Los Baños, Laguna, Philippines; b Departament de Produccio Vegetal I Ciencia Forestal, Universitat de Lleida, Lleida, Spain; c National Academy of Science and Technology Philippines, Bicutan, Taguig, Philippines; d National Institute of Molecular Biology and Biotechnology, University of the Philippines Los Baños, Los Baños, Laguna, Philippines; e Philippine Genome Center, Program for Agriculture, Livestock, Fisheries and Forestry, Office of the Vice-Chancellor for Research and Extension University of the Philippines Los Baños, Los Baños, Laguna, Philippines; f Institute of Computer Science, University of the Philippines Los Baños, Los Baños, Laguna, Philippines; g Department of Agricultural Food and Nutritional Science, University of Alberta, Edmonton, Alberta, Canada; h Department of Chemistry, University of Alberta, Edmonton, Alberta, Canada; Indiana University Bloomington

## Abstract

Ralstonia solanacearum strain T523 is the major phytopathogen causing tomato bacterial wilt in the Philippines. Here, we report the complete chromosome and draft megaplasmid genomes with predicted gene inventories supporting rhizosphere processes, extensive plant virulence effectors, and the production of bioactive signaling metabolites, such as ralstonin, micacocidin, and homoserine lactone.

## ANNOUNCEMENT

Ralstonia solanacearum causes bacterial wilt, one of the most important plant diseases worldwide (1). Bacterial wilt affects 200 species in 50 different families, including tobacco, banana, and solanaceous crops, such as potato and tomato (2). Widespread outbreaks in the Philippines have affected various economically important crops, with severe effects on tomato production (3). Here, we report the genome of R. solanacearum strain T523, isolated from wilting tomatoes in the Philippines (3).

Genomic DNA was extracted from R. solanacearum strain T523 cells grown in Kelman’s tetrazolium chloride medium (24 h, 28°C) using an MG genomic DNA purification kit (MGmed-Doctor Protein, Republic of Korea), according to the manufacturer’s protocol. The whole genome was sequenced at Macrogen, Inc. (Republic of Korea), from 10 µg of genomic DNA using a PacBio P6 DNA polymerase binding kit and a PacBio version 4.0 sequencing kit with eight single-molecule real-time (SMRT) cells (C4 chemistry) on the PacBio RS II platform. This generated 139,215 reads from a 20-kb SMRT library (mean subread length, 6,474 bp; *N*_50_, 9,102 bp). The 9.01-Mb reads were *de novo* assembled into contigs using the Hierarchical Genome Assembly Process (HGAP version 2.3) (4) to generate a final genome of 5,722,229 bp. One contig is a complete, closed, circular chromosome with a size of 3,652,934 bp, a G+C content of 67%, and a coverage of 98×. A second contig is the megaplasmid, with a size of 2,069,295 bp, a G+C content of 67%, and a coverage of 112×. Gene prediction was performed independently using the NCBI Prokaryotic Genome Annotation Pipeline (PGAP) (5) and the Joint Genome Institute-Integrated Microbial Genomes and Microbiomes (JGI-IMG/M) pipeline (6). Species identity was determined from the genome-wide average nucleotide identity (gANI) and alignment fraction (AF) using the Microbial Species Identifier (MiSI) calculator employed in IMG/M (7). Strain identity was ascertained by the digital DNA-DNA hybridization score using the Genome-to-Genome Distance Calculator (GGDC) version 2.1 (8). Bioactive secondary metabolites and virulence-associated genes were predicted using the antiSMASH version 4 (9) and Ralsto T3E (10) servers, respectively. All programs were run with default parameters unless otherwise noted.

The T523 genome has an ANI of >99% (AF, 0.9) and a dDDH (formula 2) of <70% with R. solanacearum GMI1000 and other phylotype I strains, thereby supporting the nomenclature. The genome revealed an extensive repertoire of biosynthetic gene clusters and type III virulence effectors supporting rhizosphere processes and plant symbiotic associations. The chromosome encodes a complete gene cluster for micacocidin biosynthesis, a siderophore utilizing a hybrid pathway of nonribosomal peptide synthetase, and a type I iterative polyketide synthase (11). The megaplasmid encodes genes involved in the production of the antibiotic lipopeptide ralstonin/ralsolamycin, with established phytotoxic (12, 13) and antifungal (12, 14) activities, and a putative bacteriocin. Biosynthetic gene clusters for exopolysaccharide, terpene, and homoserine lactone production were detected. Virulence-associated enzyme loci were identified, including pectinase, cellulase, and phospholipase C. Finally, the Ralsto T3E server predicted 37 and 45 *rip 77* (*Ralstonia*-injected proteins) genes (10) located in the chromosome and megaplasmid, respectively.

### Data availability.

The sequences were deposited in DDBJ/ENA/GenBank under accession numbers CP022702 and CP022703 for the chromosome and megaplasmid, respectively. The sequencing reads were deposited in the SRA under the accession number SRP159038.
